# A Virtual Coach (Motibot) for Supporting Healthy Coping Strategies Among Adults With Diabetes: Proof-of-Concept Study

**DOI:** 10.2196/32211

**Published:** 2022-01-21

**Authors:** Giulia Bassi, Claudio Giuliano, Alessio Perinelli, Stefano Forti, Silvia Gabrielli, Silvia Salcuni

**Affiliations:** 1 Department of Developmental and Socialization Psychology University of Padova Padova Italy; 2 Digital Health Lab, Centre for Digital Health and Wellbeing Fondazione Bruno Kessler Trento Italy; 3 CIMeC, Center for Mind/Brain Sciences University of Trento Rovereto Italy

**Keywords:** virtual coach, diabetes mellitus, adults, psychosocial factors, mindfulness, proof-of-concept study, mobile phone

## Abstract

**Background:**

Motivation is a core component of diabetes self-management because it allows adults with diabetes mellitus (DM) to adhere to clinical recommendations. In this context, virtual coaches (VCs) have assumed a central role in supporting and treating common barriers related to adherence. However, most of them are mainly focused on medical and physical purposes, such as the monitoring of blood glucose levels or following a healthy diet.

**Objective:**

This proof-of-concept study aims to evaluate the preliminary efficacy of a VC intervention for psychosocial support before and after the intervention and at follow-up. The intent of this VC is to motivate adults with type 1 DM and type 2 DM to adopt and cultivate healthy coping strategies to reduce symptoms of depression, anxiety, perceived stress, and diabetes-related emotional distress, while also improving their well-being.

**Methods:**

A total of 13 Italian adults with DM (18-51 years) interacted with a VC, called Motibot (motivational bot) using the Telegram messaging app. The interaction covered 12 sessions, each lasting 10 to 20 minutes, during which the user could dialogue with the VC by inputting text or tapping an option on their smartphone screen. Motibot is developed within the transtheoretical model of change to deliver the most appropriate psychoeducational intervention based on the user’s motivation to change.

**Results:**

Results showed that over the 12 sessions, there were no significant changes before and after the intervention and at follow-up regarding psychosocial factors. However, most users showed a downward trend over the 3 time periods in depression and anxiety symptoms, thereby presenting good psychological well-being and no diabetes-related emotional distress. In addition, users felt motivated, involved, encouraged, emotionally understood, and stimulated by Motibot during the interaction. Indeed, the analyses of semistructured interviews, using a text mining approach, showed that most users reported a perceived reduction in anxiety, depression, and/or stress symptoms. Moreover, users indicated the usefulness of Motibot in supporting and motivating them to find a mindful moment for themselves and to reflect on their own emotions.

**Conclusions:**

Motibot was well accepted by users, particularly because of the inclusion of mindfulness practices, which motivated them to adopt healthy coping skills. To this extent, Motibot provided psychosocial support for adults with DM, particularly for those with mild and moderate symptoms, whereas those with severe symptoms may benefit more from face-to-face psychotherapy.

## Introduction

### Background

Physical, medical, and psychosocial factors significantly contribute to adherence rates to the clinical recommendations in adults with diabetes mellitus (DM) by promoting or hindering optimal diabetes self-management. Appropriate diabetes self-management is central to long-term diabetes care, and it includes several healthy behaviors, such as monitoring of glycemic levels, physical exercise, healthy eating, taking prescribed medication and/or insulin injections, which in turn have an impact on the general well-being of people with DM. However, these healthy behaviors are difficult to maintain. Indeed, studies have shown that high levels of diabetes-emotional distress are associated with a worsening of self-care behaviors as well as glycemic levels [1]. Diabetes-emotional distress is also a risk factor for stress, anxiety, and depression symptoms. Indeed, the prevalence rates of depression are much higher in people with DM than in the general population, in which they are estimated to be 17% [1]. With regard to anxiety symptoms, studies have found that 14% of adults with DM show generalized anxiety disorder, a prevalence much higher than the 3% to 4% rate identified in a community sample [2-4]. Anxiety is related to unhealthy lifestyle choices, such as augmented smoking prevalence, assumption of food high in cholesterol, and a sedentary lifestyle, which can all lead to poor disease management [5]. In addition, higher levels of anxiety hinder cognitive capacity, which in turn influences diabetes management and thus the ability to fully follow clinical recommendations [1,5]. Similarly, feeling stressed determines the release of stress hormones, such as cortisol and adrenaline, which prevent insulin from working properly (ie, insulin resistance) and thus increases glycemic levels [6]. Depression, anxiety, and stress are associated with the risk of developing cardiovascular diseases, and the presence of DM further increases this risk [1,5,6]. All together, these factors provoke lower adherence rates and impairment in the well-being of people with DM, leading to poor disease outcomes [7]. Therefore, one can assume the presence of a complex interplay between psychosocial factors and diabetes management, meaning that they influence one another. Thus, the American Association of Diabetes Educators (AADE) guidelines introduced a healthy coping construct to identify healthy coping strategies to reduce these symptoms and improve the general well-being of adults with DM [8]. In particular, AADE suggests strategies to cope with life stresses and the challenges of managing DM, such as meditating [8]. Indeed, several studies have demonstrated the efficacy of mindfulness practices in emphasizing self-acceptance in the general population [9] and treating depressive symptoms in people with DM [10,11]. In this context, motivation is a core component in adherence to the diabetes regimen as it is specifically conceptualized for its process rather than for a specific goal [12]. Indeed, the transtheoretical model of change (TTMC) [13] defines motivation as a continuum rather than as an all-or-nothing construct, in which the individual can move across 5 stages (ie, precontemplation, contemplation, preparation, action, and maintenance), thus moving forward or backward. In this regard, digital health technologies play a central role in promoting health care, especially in chronic diseases. For instance, TTMC has been widely used in digital solutions to predict or evaluate behavioral changes in physical activity [14], diet [15], and glycemic control [16] and to ameliorate adherence to medications in adults with risk factors for the onset of cardiovascular diseases [17,18].

### Virtual Coaches: User Engagement and User Experience

The increasing rates of diabetes worldwide are a problem for the diabetologists treating diabetes, who already, as things stand, do not have enough time to assist every patient with physical, medical, and psychosocial issues. In this regard, virtual coaches (VCs) have become an increasingly relevant resource for the management of chronic diseases and for promoting behavioral changes in the self-management of individuals. Indeed, they aim to provide personalized guidance and improve intervention outcomes by mimicking human beings [19]. Indeed, VC in the health care field is mainly aimed at developing personalized user-system interactions and supporting individuals in their behavioral changes [20,21]. This is important for improving user engagement (UE) and compliance, both of which are crucial for achieving long-term behavioral changes and adjustment toward a healthier lifestyle [22]. UE is a multifaceted construct that refers to the quality of the user experience (UX), including the individual’s time, cognitive, affective, and behavioral investment during the interaction with a digital solution [23]. The UE construct goes beyond user satisfaction: indeed, the literature suggests that the capacity to engage and maintain engagement in the interaction with a digital solution seems to show positive results in eHealth, e-learning, and web searching [23]. Indeed, prolonged engagement has been shown to be promising in a diabetes prevention program with the use of VC [24], engaging 69% of adults for the whole study and resulting in 8.98% weight loss [24]. In addition, in 2 other recent studies, adults with type 2 DM (T2DM) [25] and young adults with type 1 DM (T1DM) [26] reported feeling engaged and satisfied with a VC embedded in an application. In other studies, people with DM reported an increased level of satisfaction with the interaction with the VC [27,28]. Indeed, VCs for people with DM seem to favor self-care behaviors and behavioral changes as well as support them at follow-up. For instance, a recent review reported that VCs for people with DM represent effective interventions for fostering their glycemic control, in combination with standard care [29]. Therefore, VCs seem to be capable of overcoming common barriers related to adherence by delivering data-driven personalized support in real time and being available at any time during the day [30], thus allowing scalability. In this regard, UX is a crucial element that intersects the UE. Taking into consideration the definition proposed by the International Organization for Standardization [31], UX includes users’ engagement, pleasure, desirability, values, emotions, beliefs, preferences, perceptions, physical and psychological responses, behaviors, and accomplishments, which occur before, during, and after the use of a digital solution. The International Organization for Standardization also lists 3 factors that influence UX: the user’s current state and previous experience, system properties, and use context [31]. Therefore, understanding users’ needs, their working environments, interactions, and emotional reactions can help design VCs from the UX point of view [31]. Thus, the user becomes an active contributor to this process [31]. Studies have shown that higher levels of UX have been associated with an increased effectiveness of digital health interventions targeting improvements in T2DM self-management [25], physical activity [32], and diet [33]. However, there is a lack of evidence regarding UE and UX as constructs that interplay in the development, evaluation, and implementation of VCs for psychosocial support of adults with DM.

### Comparison With Previous Work

To our knowledge, this is the first study to implement VC for psychosocial support in adults with T1DM and T2DM. Notwithstanding the originality of this work, in previous studies, VCs have been deployed to improve healthy coping strategies in college students, showing their beneficial effect in reducing symptoms of distress [34,35]. With regard to the development of VCs in the field of diabetes, studies have designed a conversational agent [36] and an interactive diary [37,38]. These digital interventions were both embedded in a smartphone app to improve health-related quality of life among adults with T2DM [35] and T1DM [37,38]. Health-related quality of life is an important and well-known construct that underlies the concept of general well-being. However, it should also be noted that anxiety, depression, and stress symptoms interplay with diabetes management. This means that these outcomes can hinder an individual’s ability to manage diabetes and maintain effective glycemic control. Hence, it is important to include these variables when developing programs and interventions for adults with DM.

### Objectives

Bearing all these aspects in mind, this VC (Motivational bot—Motibot) aims to support and motivate adults with T1DM and T2DM to adopt healthy coping strategies. In turn, these healthy coping strategies should reduce depression, anxiety, perceived stress symptoms, and diabetes-related emotional distress and improve well-being. Therefore, the aim of this proof-of-concept study was threefold:

1. To evaluate the preliminary efficacy of the VC intervention before and after the intervention and at follow-up in reducing the abovementioned psychosocial symptoms while also improving the well-being of adults.
2. To investigate UX and UE with the VC for psychosocial support accessed through personal smartphones within the Telegram messaging app.
3. To evaluate semistructured interviews on both UX and how users felt during their interaction with the VC.

## Methods

### Participants and Recruitment

The study involved 18 voluntary adults with T1DM and T2DM recruited in Italy via social network sites (ie, Facebook groups) using snowball sampling. Five adults dropped out of the study for personal and medical reasons. Therefore, the final sample included 13 adults aged between 18 and 51 years (mean 30.08, SD 10.61 years), 77% (10/13) of which were women; 62% (8/13) of adults had T1DM and 39% (5/13) had T2DM, with an overall mean diabetes duration of 10 (SD 8.49) years. One participant did not complete the psychological measures after the intervention and was therefore excluded from the analyses of the psychosocial variables. The inclusion criteria for participating in this study were as follows: (1) having T1DM or T2DM and (2) owning a smartphone and a Telegram account. The decision to include both types of DM was guided by the notion that there are similarities between the lifestyle guidelines for adults with T1DM and T2DM, as emerged from the results of a recent meta-analysis [39]. Participants were excluded if they had gestational diabetes or prediabetes.

### Procedure and Ethics

This work is a proof-of-concept study for adults with T1DM and T2DM, conducted following the Obesity-Related Behavioral Intervention Trials (ORBIT) framework [40]. The ORBIT model supported guidance throughout the whole process as it emphasizes the importance of adopting a data-driven iterative approach to optimize subsequent iterations of the intervention [40]. In particular, this model places the user at the center of the design process. The study was conducted in compliance with the Declaration of Helsinki (Italian law 196/2003, European Union General Data Protection Regulation 679/2016). The Interdepartmental Ethical Committee of Psychology of the University of Padova (Italy) approved the project (approval number: 3968; February 3, 2021), stating that there were no critical ethical issues. The participants signed a written informed consent sent via mail, agreeing to participate in the study and semistructured interviews 1 month after the end of the study. They were informed that their data would be confidential, that they could omit any information they did not wish to give, and that they could withdraw from the study at any moment without having to provide any explanation.

### Intervention Description: Motibot Design

**Figure 1:**
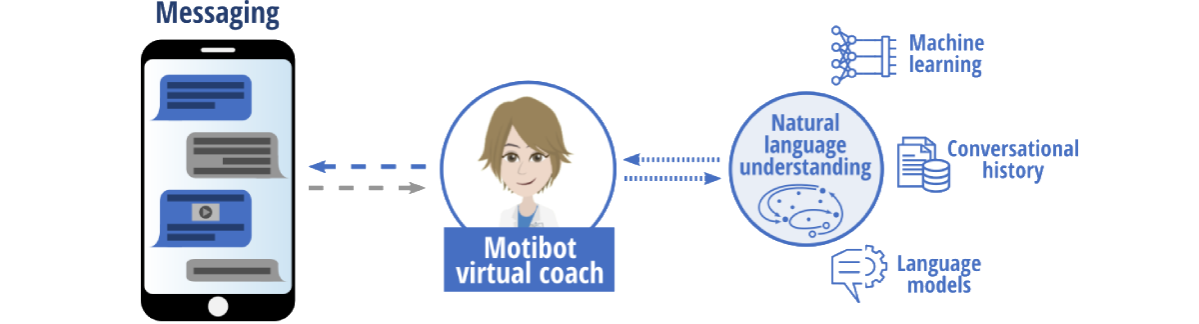
Motibot: the virtual coach.

**Figure 2:**
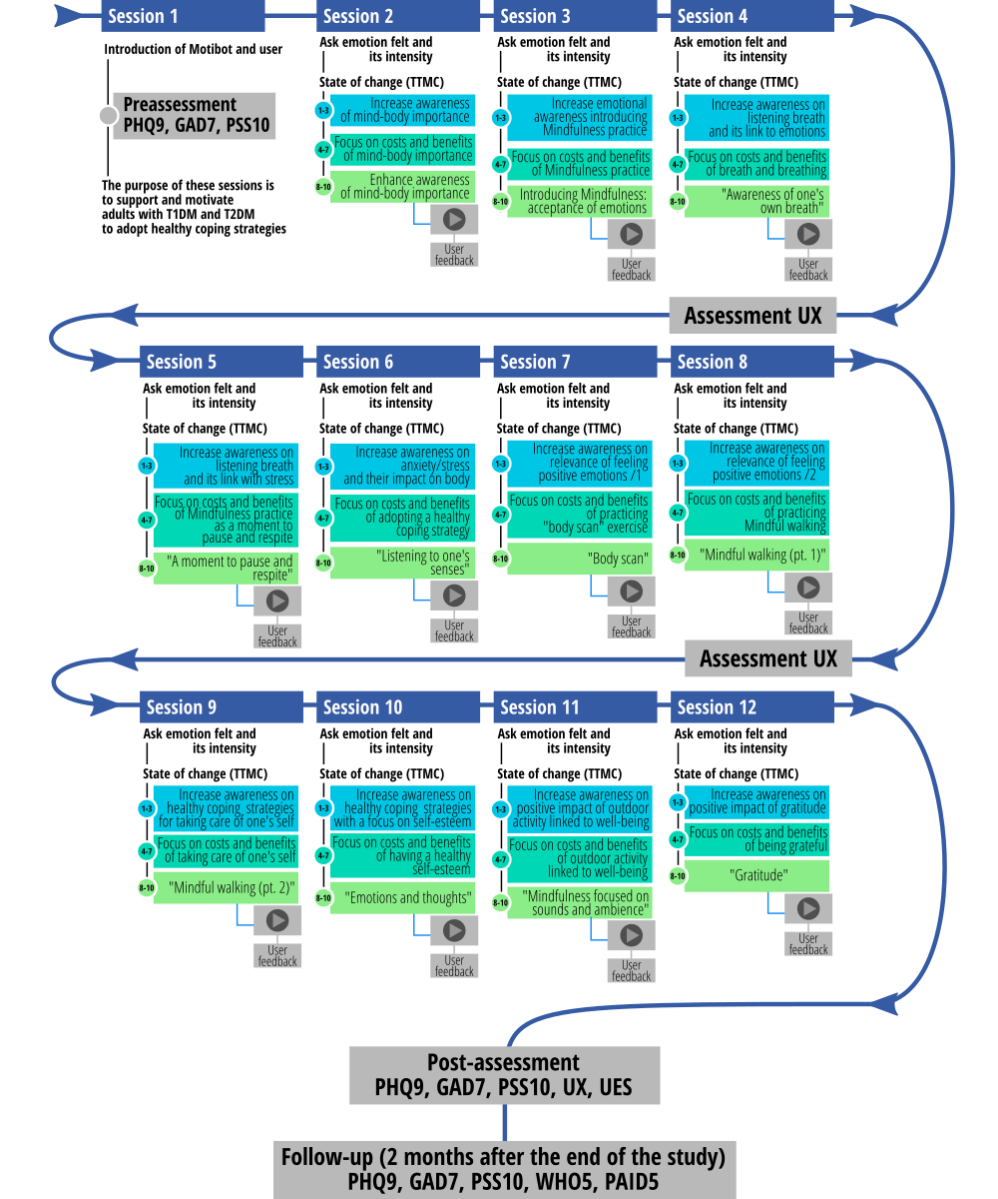
Graphical representation of the conversational protocol delivered to users and its chronological structure. GAD7: Generalized Anxiety Disorder-7 Items; PAID5: Problem Areas in Diabetes Scale–Short Form-5 Items; PHQ9: Patient Health Questionnaire-9 Items; PSS10: Perceived Stress Scale-10 Items; T1DM: type 1 diabetes mellitus; T2DM: type 2 diabetes mellitus; TTMC: transtheoretical model of change; UES: User Engagement Scale; UX: user experience; WHO5: The World Health Organization-5 Well-Being Index.

*Motibot* (Figure 1) is a VC designed to provide psychosocial support by motivating adults with DM to adopt and cultivate healthy coping strategies, which, according to the AADE guidelines, should be flexible and adaptable to the users’ needs [8]. These coping strategies, in turn, should foster adults’ well-being by reducing depression, anxiety, and perceived stress symptoms and diabetes-related emotional distress. Motibot was developed by the Digital Health Lab at Fondazione Bruno Kessler Research Center for Digital Health and Wellbeing using Rasa [41], an open-source platform designed for the development and training of VCs. It was then deployed through the Telegram messaging app. The environment provided by Rasa exploits machine learning (ML) libraries and pretrained embeddings from language models, thus allowing the construction of a VC for a specific language by combining ML approaches and handcrafted rules. Motibot relies on natural language understanding (NLU) [42], which is an ML technique that enables the VC to interpret user messages. NLU, together with the conversational history and a set of predefined variables, determines the transition from one turn of the dialogue to another. In this study, the NLU system was trained by feeding it with a data set comprising 6899 examples of user utterances categorized by intent and annotated with entities. Examples of intents are to *affirm, deny, say your name, say what you feel, schedule the next meeting*, and *xpress the level of motivation*e. Examples of entities are the *user’s name, the emotion felt, the date, time of the next meeting*, and *level of motivation*. NLU was used to interpret the intents and entities. In this study, we defined 54 intents and 6 entities. During interactions between Motibot and users, intents and entities were extracted from users’ messages and classified using a trained multitask transformer architecture. Motibot was designed to last for 12 sessions of 10-20 minutes each, during which Motibot interacted with the user according to the scripts previously defined as shown in Figure 2. Because of the flexibility of this VC, specifically designed to be as adaptable as possible to the users’ daily life, every session was initiated following a scheduled plan decided by the users themselves to best suit their needs. Users were able to respond to Motibot by inputting text or tapping an option on their smartphone screen. The interaction between Motibot and users is designed considering evidence-based approaches related to counseling and psychoeducation as displayed in Figure 2. In particular, these approaches are linked to the healthy coping construct [8] and to mindfulness-based cognitive therapy [43] to support and motivate the development and/or enhancement of coping strategies. For these reasons, the whole conversational protocol was developed referring to TTMC [13], which allows the VC to understand what motivational state the user is in and consequently deliver the most appropriate psychoeducation intervention, which is based on the user’s motivation to change. At the beginning of the first session, Motibot asks the users to present themselves by telling they are. Subsequently, Motibot delivers a video presentation of itself, its functionality, and its main features to involve the user in the interaction. Thereupon, Motibot delivers 3 different questionnaires to assess the levels of depression (Patient Health Questionnaire-9 [PHQ-9]) [44], anxiety (Generalized Anxiety Disorder-7 [GAD-7]) [45], and perceived stress symptoms (Perceived Stress Scale-10 [PSS-10]) [46]. These 3 questionnaires were also sent after the intervention and at follow-up. In this last case, 2 psychosocial scales were added to assess diabetes-related emotional distress (Problem Areas in Diabetes Scale–Short Form-5 [PAID-5]) [47] and general well-being (World Health Organization-5 Well-Being Index [WHO-5]) [48]. PAID-5 and WHO-5 were evaluated only at follow-up, that is, 2 months after the end of the study. The latter 2 scales were included to verify whether coping strategies had been internalized, thus leading to greater well-being. Indeed, diabetes self-management is well known to be influenced by these outcomes. In addition, Motibot sent 2 other questionnaires to assess UX during the whole interaction and UE only at the end of the intervention to comprehend the users’ overall and final involvement. One month after the end of the study, semistructured interviews were conducted to understand both UX and how users felt during their interaction with Motibot.

Each day, Motibot asks the users what emotion they are feeling at that precise moment as well as its intensity, to support them in becoming more aware of their own emotions and self-reflecting on them. After this question, according to TTMC and following the state of change ruler (ie, precontemplation state, contemplation, preparation, action, and maintenance) [13], Motibot asks users “How much do you want to improve your well-being on a scale from 1 (not at all) to 10 (very much)?” to understand their motivation to maintain diabetes under control. When users are considered in the *precontemplation state*, Motibot tries to investigate why they feel this way and then attempts to increase emotional awareness by helping them to self-reflect on their emotions and on the importance of taking care of both their body and mind. When users are considered in the *contemplation state*, Motibot provides motivational interventions, which focus the attention on the costs and benefits of adopting a healthier behavior to favor psychosocial well-being. Finally, when users are in the *action state*, Motibot provides behavioral interventions by sending audio tracks related to mindfulness practices.

### Data Collection

As reported in Textbox 1, the psychosocial questionnaires were administered before and after the intervention and at follow-up. Moreover, semistructured interviews were conducted 1 month after the end of the study.

**Overview of questionnaires and of their administration timing.**

- Before the intervention
  Patient Health Questionnaire-9 (depression)
  Generalized Anxiety Disorder-7 (anxiety)
  Perceived Stress Scale-10 (perceived stress)
- At the 2nd, 8th, and 12th sessions
  User Experience Questionnaire (user experience)
- After the intervention
  Patient Health Questionnaire-9
  Generalized Anxiety Disorder-7
  Perceived Stress Scale-10
  User Engagement Scale–Short Form (user engagement)
- 1 month after the end of the study
  Semistructured interviews
- At follow-up (2 months after the end of the study)
  Patient Health Questionnaire-9
  Generalized Anxiety Disorder-7
  Perceived Stress Scale-10
  World Health Organization-5 Well-Being Index (well-being)
  Problem Areas in Diabetes Scale–Short Form-5 (diabetes-related emotional distress)

The *PHQ-9* [44] is a brief self-reported unidimensional measure developed to assess and monitor the severity of depression symptoms in the previous 2 weeks. The questionnaire includes 9 items rated on a 4-point Likert scale (from 0 *never* to 3 *almost every day*). The PHQ-9, which incorporates the Diagnostic and Statistical Manual of Mental Disorders, fourth edition, text revision criteria, has a total score ranging from 0 to 27, with a score of 10 representing the optimal cutoff to detect clinically relevant depression. The PHQ-9 comprises five categories of severity: (1) absent (scores 0-4), (2) subthreshold depression (scores 5-9), (3) mild depression (scores 10-14), (4) moderate depression (scores 15-19), and (5) major depression (scores 20-27). An example of an item is the following: “During the last two weeks, on how many days did you feel little interest or pleasure in doing things?” (item 1). The PHQ-9 has shown good psychometric properties [44]. The *GAD-7* [45] is a brief self-reported unidimensional measure aimed at screening probable cases of GAD and assessing the severity of symptoms in the previous 2 weeks. The questionnaire comprises 7 items based on a 4-point Likert scale (from 0 *never* to 3 *almost every day*). The GAD-7 incorporates the Diagnostic and Statistical Manual of Mental Disorders, fourth edition, text revision criteria and has a total score ranging from 0 to 21, with a score of 10 as the cutoff for GAD. The questionnaire included three categories of severity: (1) mild anxiety symptoms (score ≥5), (2) moderate anxiety symptoms (score ≥10), and (3) severe anxiety symptoms (score ≥15). An example of an item is the following: “In the last two weeks, how often did each of the following problems bother you? Feeling nervous, anxious, or tense” (item 1). The GAD-7 has demonstrated good validity and reliability [45]. The *PSS-10* [46] is a brief self-reported unidimensional measure that assesses an individual’s perception of stress in the previous month. The PSS is a measure of the degree to which each situation in one’s life is perceived as stressful; indeed, the items are designed to evaluate the degree to which individuals find their lives unpredictable, uncontrollable, or overloaded. The scale also contains a series of direct questions about the current levels of perceived stress. The PSS consists of 10 items based on a 4-point Likert scale (from 0 never to 5 very often). The total PSS score ranges from 0 to 40, with high scores indicating a high level of perceived stress. PSS includes three categories of severity: (1) low perception of stress (scores 0-13), (2) moderate perception of stress (scores 14-26), and (3) high perception of stress (scores 27-40). An example of an item is the following: “In the last month, how often have you felt out of sorts because something unexpected happened?” (item 1). The PSS-10 has demonstrated good psychometric properties regarding reliability and validity [46]. The *PAID-SF-5* [47] is a self-reported unidimensional measure aimed at assessing diabetes-related emotional distress. The questionnaire comprises 5 items based on a 5-point Likert scale (from 0 *not a problem* to 4 *serious problem*). Total scores range from 0 to 100, with higher scores (ie, ≥40) indicating greater diabetes-related emotional distress. The PAID-SF-5 has demonstrated good psychometric properties [47]. The *WHO-5* [48] is a self-reported unidimensional measure that evaluates psychological well-being, a core dimension of quality of life. The questionnaire comprises 5 items rated on a 6-point Likert scale (from 0 never to 5 always). The total score was rescaled to range between 0 and 100, with a score ≤50 suggesting poor psychological well-being and a score ≤28, indicating depression, showing good psychometric properties [48]. The *User Engagement Scale–Short Form* (UES-SF) [49] is a brief self-report questionnaire aimed at assessing user engagement with a digital solution. The UES-SF includes 12 items based on a 5-point Likert scale (from 1 *strongly disagree* to 5 *strongly agree*). The UES-SF comprises 4 factors: (1) focused attention, which indicates the feeling of being immersed in the interaction (eg, “I lost myself in this experience”); (2) perceived usability, which is the negative affect experienced owing to the interaction and the effort spent (eg, “I felt frustrating while using Motibot”)—this factor is the only one in which the scores were reversed; (3) aesthetic appeal, which represents the graphical and visual appeal related to the digital solution (eg, “Motibot was aesthetically appealing”); and (4) the reward factor (eg, “Using Motibot was worthwhile”). The latter is a single set of 3 factors related to the original UES questionnaire [49,50], such as the endurability, which evaluates the overall success of the interaction; the novelty, which examines the overall interest related to the interaction with a digital solution; and finally, the felt involvement factor, which evaluates the overall fun interaction. The overall scale was found to be reliable [49]. The *User Experience Questionnaire* (UEQ) used in this study is an adapted version of the original UEQ [51], modified ad hoc to make the bipolar adjectives more appropriate to the aims of this study. In particular, the questionnaire included 28 adjectives, either positive or negative, designed to assess the experience of interacting with the VC. Each item was scored on a 5-point Likert scale (from 1 *strongly disagree* to 5 *strongly agree*). Textbox 2 shows the selection of items for this study.

**The items of the User Experience Questionnaire.**

**Positive items**

- Pleasant
- Profound
- Cordial
- Comprehensible language
- Empathetic
- Attentive
- Motivating
- Encouraging
- Supportive
- Trustworthy
- Flexible
- Interesting
- Effective

**Negative items**

- Annoying
- Not reliable
- Unappealing
- Unclear
- Complicated
- Not efficient
- Too much information
- Dissuading
- Not stimulating
- Not engaging
- Unpredictable
- Not reflective
- Conventional
- Not effective
- Rigid

*Semistructured interviews* were conducted by GB with all participants who concluded the interaction with Motibot. The interviews were based on 11 ad hoc questions administered 1 month after the end of the study and lasted approximately 10 minutes. Each interview started with asking the motivation for participating in this study and concluded with a question in which the participant should explain whether they would suggest Motibot to other people with the same chronic illness, explaining the reason. The other 9 questions were divided into 2 sections as reported in Textbox 3. The first included 5 questions related to the experience that users had with Motibot; therefore, the goal was to assess the UX. On the other hand, the second section included 4 questions related to how users felt during the interaction with Motibot from a psychological perspective.

**Questions asked to participants during semistructured interviews.**

- What motivated you to participate in the study?
  User experience
    What were your expectations with regard to Motibot?
    With regard to Motbiot, which aspect did you like the most?
    With regard to motibot, which aspect did you dislike the most?
    With regard to Motibot, how was your user experience?
    Would you be interested in using, in the future, a complete virtual coach?
- How users felt during the interaction
  Motibot proposed to you several audio tracks regarding mindfulness. How did you live them?
  Did you find Motibot useful to find a mindful moment for yourself?
  Did Motibot help you to soothe any anxiety, stress and/or depression symptoms?
  Did you listen to Motibot mindfulness audio tracks again at the end of the study?
- Would you suggest Motibot to someone with diabetes mellitus? Why?

### Statistical Analysis

Statistical analyses were performed using R, version 4.0.0 (The R Foundation for Statistical Computing) [52], and SPSS Statistics, version 24.0 (IBM Corp) [53]. The Shapiro-Wilk test was performed to evaluate the normality of the sample distributions of the variables investigated in this study. Descriptive analysis was carried out on psychological dimensions, namely depression, anxiety, and perceived stress, before and after the intervention and at follow-up. The same analysis was performed on diabetes-related emotional distress and well-being, although only at follow-up. All data are shown as plots. The Kruskal-Wallis nonparametric test was used to evaluate differences in depression, anxiety, and stress among participants. A post hoc Wilcoxon nonparametric test was performed to compare the differences in the aforementioned outcomes before and after the intervention and at follow-up to understand whether the psychoeducational intervention had been effective. Means and SDs were computed for UX, which was evaluated at the 4th, 8th, and 12th sessions and for UE, which was evaluated at the end of the study. The data regarding UX are displayed as plots. A text mining approach [54,55] was followed to extract information from the semistructured interviews on UX and on how users felt during the interaction with Motibot. This analysis was implemented by relying on the *Quanteda* R package [56] and on custom shell scripting code under a Linux environment. The analysis was carried out on the written interview transcripts (in Italian) as follows: first, they were cleaned by replacing uppercase letters and removing numbers, punctuation, and stopwords. Thereupon, user’s answers were divided into groups, each containing all answers to one of the interview questions. Two analyses steps were implemented: (1) extraction, from some of the questions in the semistructured interviews, of 3 sets of responses (ie, yes/no/maybe) and (2) extraction, from the remaining questions, of recurrent concepts (ie, word stems) and their relations in terms of digrams (ie, pairs of word stems). A word stem was deemed to be recurrent if it appeared at least three times across interviews, whereas digrams were considered recurrent if they appeared at least two times. The criterion of 3 occurrences as the threshold for including a stem was chosen according to the following rule of thumb. We assume stems to be significant if they belong to the 5% most recurrent ones. However, because occurrence is quantified by an integer number, this percentile threshold can be enforced only approximately. Setting the criterion of minimum occurrences to 3 yielded the extraction, for the different questions, between 3.8% and 7.9% most recurrent stems (average 6.2%), in reasonable compliance with the 5% threshold assumed above. In addition, the average occurrence of stems for a given question was 1.35; a threshold of 3 occurrences was equivalent to the requirement of a stem recurring more than twice as frequently as the average.

## Results

### Preliminary Analysis

The variables investigated in this study showed a nonnormal distribution. No missing data were identified, and each participant answered all the questions administered. As mentioned in the *Participant* section, only one participant did not answer the entire questionnaire sent after the intervention and thus was excluded from the analyses regarding the psychosocial variables.

### Perceived Stress, Anxiety, and Depression Symptoms

**Figure 3:**
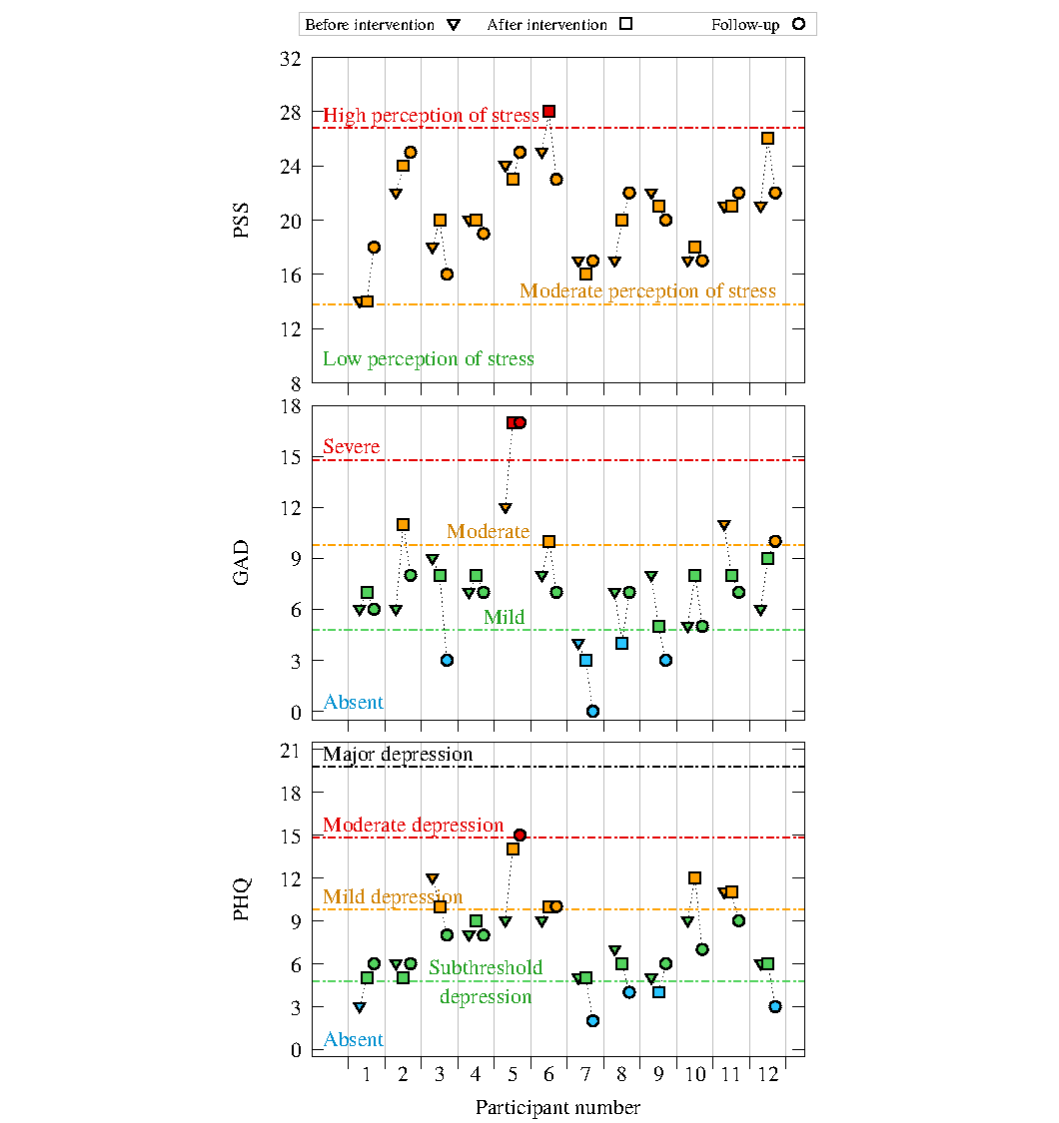
Plots of perceived stress, anxiety, and depression symptoms, assessed through the Perceived Stress Scale-10 (PSS-10), Generalized Anxiety Disorder-7 (GAD-7), and Patient Health Questionnaire-9 (PHQ-9), respectively, before and after intervention and at follow-up (N=12).

Overall, as displayed in Figure 3, all participants showed moderate symptoms concerning perceived stress (assessed using the PSS-10). Participant 6 was the only participant showing a high perception of stress after the intervention; however, the level of perceived stress diminished at follow-up. Data concerning anxiety and depression symptoms (assessed respectively through the GAD-7 and PHQ-9) seem to increase after the intervention and decrease at follow-up, except for participant 5, who presented severe symptoms at both time points, thereby resulting in an outlier. The presence of an outliers did not affect the overall trend of data regarding these outcomes.

### Well-being and Diabetes-Related Emotional Distress

**Figure 4:**
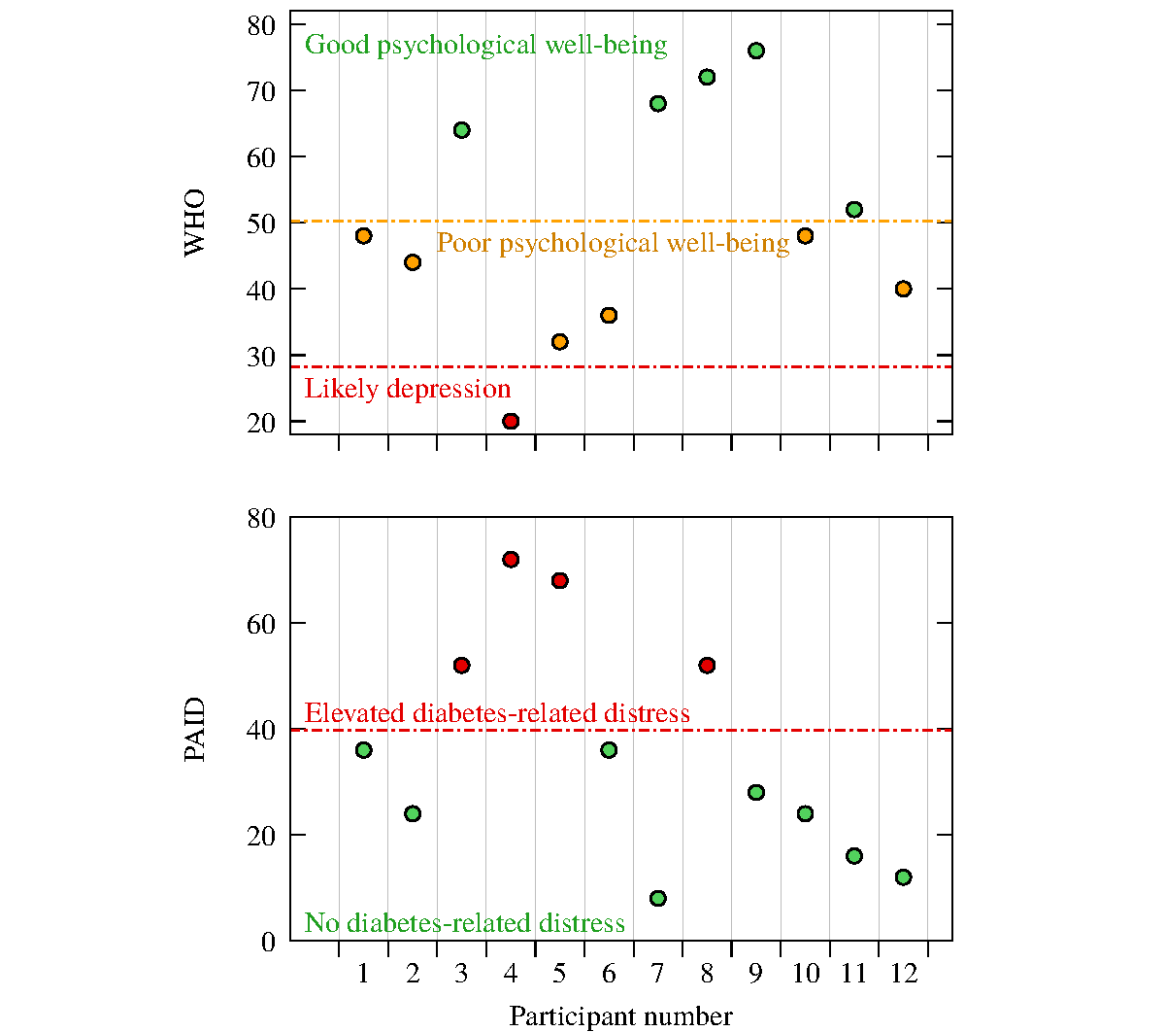
Plots of well-being and diabetes-related emotional distress, evaluated through the World Health Organization-5 Well-Being Index (WHO-5) and Problem Areas in Diabetes Scale-5 (PAID-5), respectively, at follow-up (N=12).

As shown in Figure 4, when considering the presence of an outlier, participants ranged between poor and good psychological well-being (assessed through the WHO-5), with an overall mean of 50.00 (SD 17.18), which indicates an overall poor psychological well-being. However, if the outlier is excluded, the overall mean is 51.64 (SD 17.01), which corresponds to an overall good psychological well-being. With regard to diabetes-related emotional distress (assessed through the PAID-5), most participants did not present diabetes-related emotional distress; indeed, the overall mean was 35.67 (SD 21.20). If the approach described above is applied and therefore if the outlier is excluded, the overall mean is 32.73 (SD 19.50), which, being an even smaller value, suggests low levels of diabetes-related emotional distress.

### Kruskal-Wallis Test for Psychosocial Outcomes

The Kruskal-Wallis test, carried out to assess the differences among depression, anxiety, and perceived stress symptoms, did not yield any significant results when considering the intervention period. Nevertheless, as shown in Figure 3, a downward trend can be identified over the 3 time periods (ie, before the intervention, after the intervention, and during follow-up).

### User Experience

**Figure 5:**
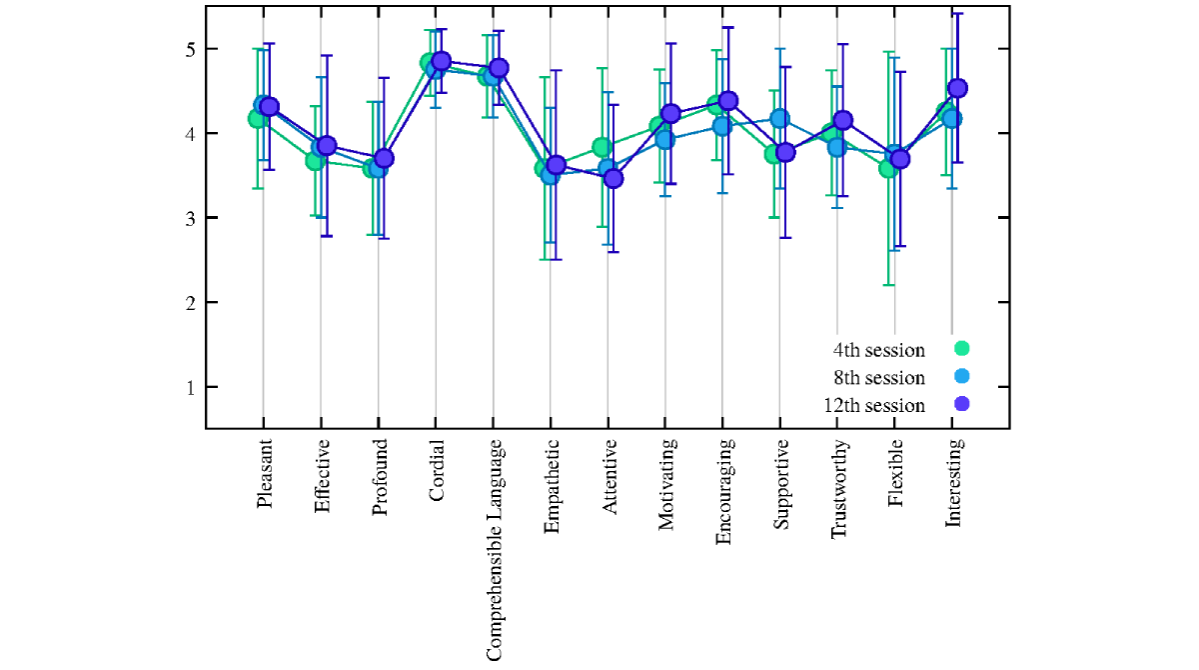
Plot of the positive items of the User Experience Questionnaire. Circled dots and error bars correspond to sample means and sample SDs, respectively (N=13).

Analyses regarding the positive items (assessed through UEQ) reported a mean >3 on a 5-point Likert scale (mean 4.04, SD 0.22). In particular, the items *comprehensible language*, *empathetic*, *motivating*, *encouraging*, and *interesting* increased from the 2nd to the 12th session, whereas the item *supportive* tended to decrease from the 2nd to the 12th session as displayed in Figure 5. The specific means and SDs are reported in the Multimedia Appendix 1 (Table S1).

**Figure 6:**
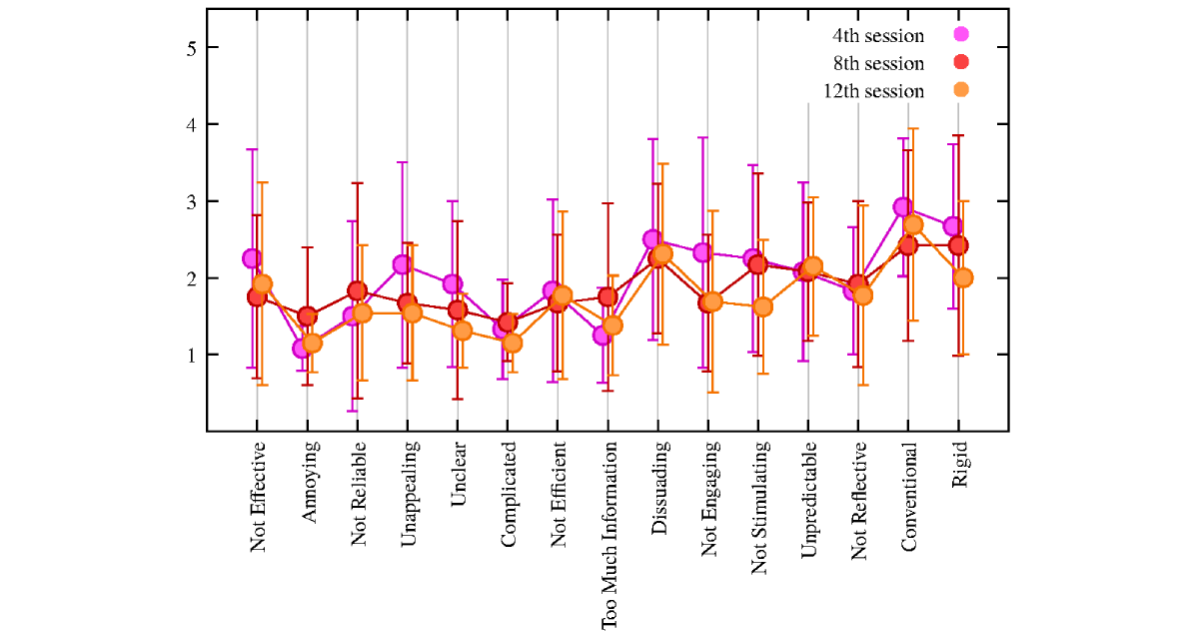
Plot of the negative items of the User Experience Questionnaire. Circled dots and error bars correspond to sample means and sample SDs, respectively (N=13).

Analyses of the negative items (evaluated using UEQ), shown in Figure 6, reported a mean <2 on a 5-point Likert scale (mean 1.86, SD 0.30), thereby attesting that users disagreed with the overall items. In particular, the items *not stimulating*, *not engaging*, and *rigid* decreased from the 2nd to the 12th session. The specific means and SDs are reported in the Multimedia Appendix 1 (Table S2).

### User Engagement

Overall, the data on UE show that participants were engaged with Motibot, as reported in Table 1. The reward factor, which refers to the worthwhile and absorbing experience of the user with the digital solution, presents a maximum value of 5. The same result also emerged for perceived usability and focused attention. Notably, the perceived usability factor is the only factor in which the items were reversed, indicating a good effect experienced by the digital solution.

**Table 1:** Descriptive statistics for User Engagement Scale (N=13).

Parameters	Value, range	Value, mean (SD)
Total scale	3.25-4.83	4.14 (0.49)
Perceived usability	4-5	4.82 (0.32)
Focused attention	2.33-5	3.62 (0.83)
Esthetic appeal	2.67-4.67	3.79 (0.55)
Reward factor	3-5	4.33 (0.58)

### Minimum and Maximum Scores of Participants Based on the User Engagement Questionnaire’s Likert Scale

#### Text Mining

**Figure 7:**
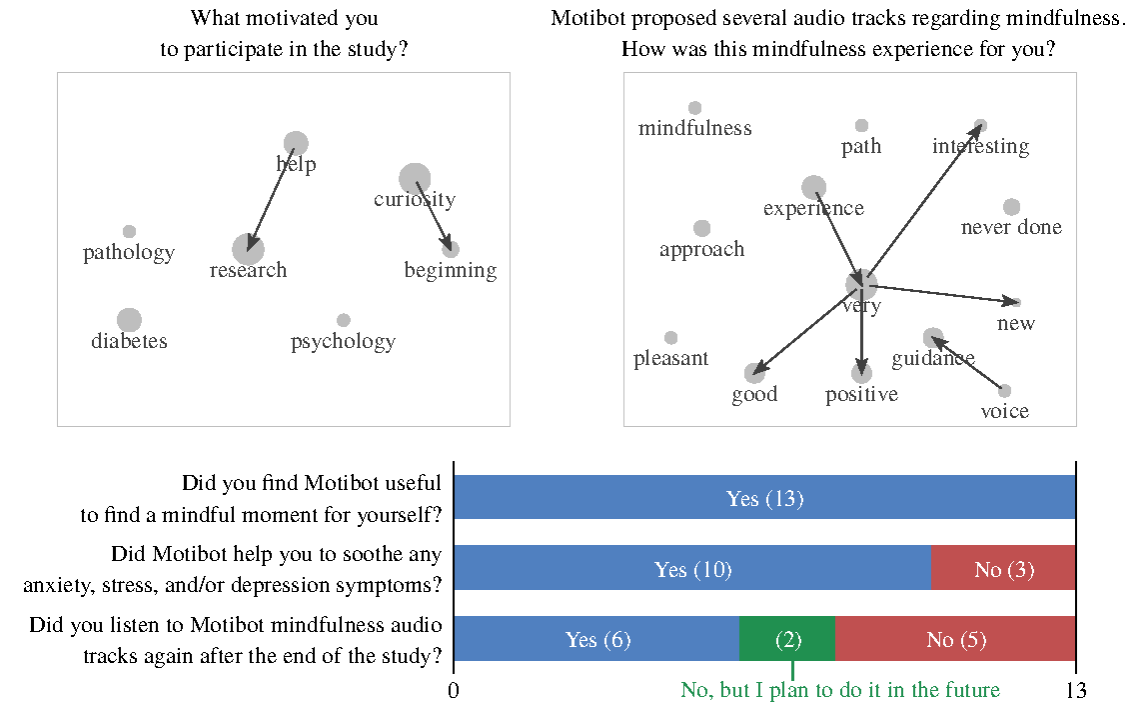
Answers to the semistructured interviews related to user experience.

**Figure 8:**
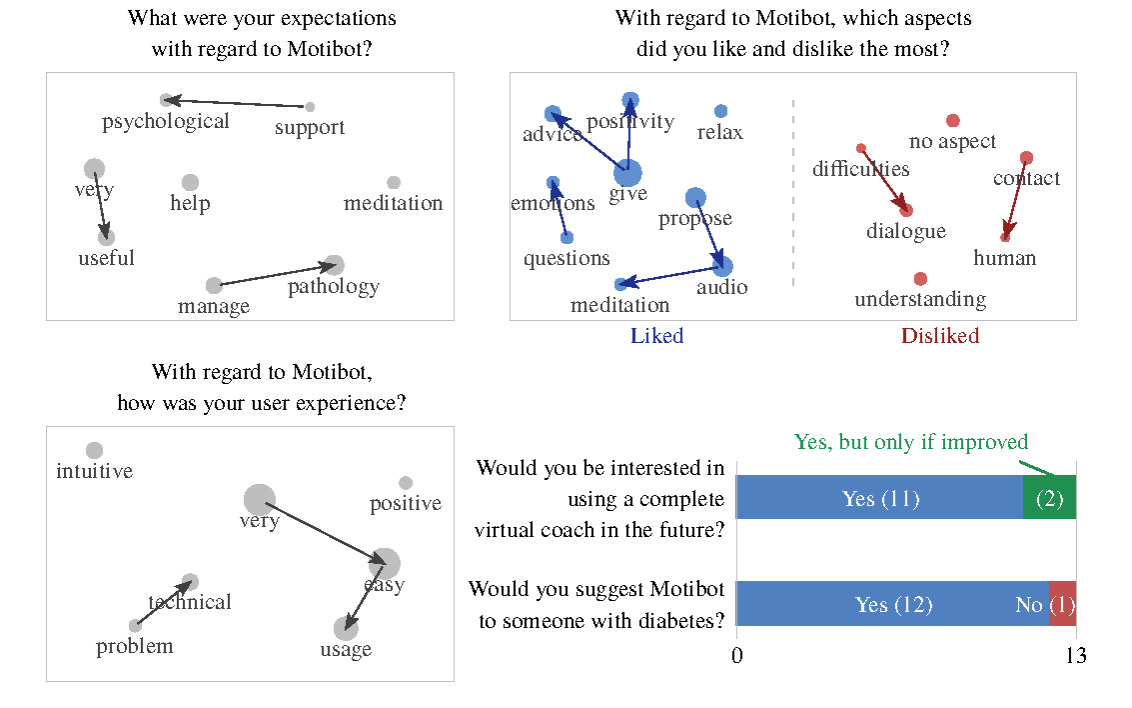
Answers to the semistructured interviews related to how users felt during the interaction with Motibot.

Overall, the average duration of the 13 semistructured interviews was 9.04 minutes. The transcripts of the interview answers comprised 562 words on average. The results of text mining applied to the answers of the semistructured interviews, concerning UX and how users felt during the interaction with Motibot, are graphically summarized in Figures 7 and 8, respectively. In both figures, bar plots show the distribution of 3 types of answers (ie, yes/no/maybe), whereas scatter plots highlight the most frequent concepts, namely word stems appearing at least three times in the interviews. Within scatter plots, arrows identify recurrent digrams, that is, sequences of 2-word stems appearing at least twice within the interviews. It is worth mentioning that the apparently opposite ordering of some digrams (eg, *support→psychological*) is because of the analysis being carried out on texts in Italian, in which word ordering is different from English. Stems were translated at the end of the analysis, considering the abovementioned potential nuances between the 2 languages. The radius of the circle is proportional to the number of occurrences of each stem.

#### Text Mining: UX With Motibot

As shown in Figure 7, 85% (11/13) of the participants would be interested in using a VC for psychosocial support and 92% (12/13) would suggest VC to other people with the same chronic disease. Overall, participants reported having a positive experience with Motibot. As displayed in the upper-right panel, where stems graphed in blue and red correspond to *liked* and *disliked* aspects, respectively, users largely reported positive aspects in the interaction with Motibot, except for some technical problems. It should be mentioned that 3 participants reported that there were no aspects that they disliked.

#### Text Mining: How Users Felt During the Interaction With Motibot?

As displayed in Figure 8, users reported mindfulness audio tracks as a very good, positive, interesting, and new experience. They further considered the voice of the audio tracks as a guide to the mindfulness pathway. Indeed, 62% (8/13) of the participants also listened to the mindfulness audio again after the end of the study or planned to do so in the future, to grant themselves a further mindful moment. Finally, 77% (10/13) of the participants reported that Motibot helped them reduce the symptoms of anxiety, depression, and/or stress.

## Discussion

### Principal Findings

This proof-of-concept study evaluated the preliminary efficacy of a VC intervention for psychosocial support. Motibot, indeed, aims to support and motivate adults with T1DM and T2DM to adopt healthy coping strategies. In turn, these healthy coping strategies should reduce depression, anxiety, perceived stress symptoms, and diabetes-related emotional distress and improve well-being. This study further aims to evaluate UX and UE in the interaction with a VC from both qualitative and quantitative perspectives. Overall, preliminary evidence suggests that the digital intervention led to improvements in symptoms of anxiety and depression, as emerged from the downward trend of the related factors detected over the 3 time periods (ie, before the intervention, after the intervention, and during follow-up). Notably, participants showed an increase in anxiety and depression symptoms after the intervention and a subsequent decrease during follow-up, showing good psychological well-being, upon exclusion of an outlier, and no diabetes-related emotional distress. These data highlight how the effects of the psychoeducational intervention were maintained over time, thus leading to the users’ internalization of healthy coping strategies and self-reflection of their own emotions. With regard to perceived stress, users showed moderate symptoms, which remained throughout the intervention, including follow-up. However, users did not present any diabetes-related emotional distress. These findings shed light on the possible stressful events that underpin perceived stress, such as the impact of contingent events; thus, the findings did not relate to the burden of managing DM. It is worth mentioning that this study was carried out in 2021 during the COVID-19 pandemic, which had a significant impact on psychological well-being among the whole population [57]. Overall, the users perceived a decrease in any symptoms of anxiety, depression, and/or stress, reporting the usefulness of Motibot in supporting and motivating them to find a mindful moment for themselves. The psychoeducational intervention was well accepted by users, particularly in the presence of a mindfulness *pathway*. Indeed, users reported a *very good*, *positive*, *interesting*, and *new experience*: most users listened to the audio tracks even at the end of the study to achieve a mindful moment for themselves once again. Indeed, mindfulness-based interventions have recently become more relevant in the context of DM care, as they are associated with a reduction of negative emotions and an enhancement of an individual’s attitude and coping strategies [58]. Users had a *positive* and *interesting experience* with Motibot, particularly because it proposed audio tracks relating to meditation and asked them what emotion they were feeling in that precise moment. The purpose of asking users to express their emotions is to motivate them to become more aware and reflect on them: one might expect that the more one is aware of their own emotions, the better they can regulate them. Motibot was perceived as *empathetic* and *stimulating* in its dialogic interaction, even if it was slightly less *supportive* from the 2nd to the 12th session. This last result might indicate that users become familiar with Motibot throughout the sessions and thus do not perceive any further support, albeit still feeling involved and absorbed in the interaction. However, Motibot was also perceived as *motivating* and *encouraging* in the adoption of healthy coping strategies: users appreciated that Motibot gave them *positive advice*. Notwithstanding these promising results, few users still reported a desire for *human contact* to receive psychological support. These data emerged particularly for those who presented with high levels of anxiety, depression, and/or perceived stress symptoms. Therefore, we speculate that VCs may be successfully used to support and motivate people with mild and moderate psychological symptoms, whereas those with more severe psychological symptoms may benefit more from psychotherapy support in face-to-face spontaneous and human settings. Furthermore, users encountered technical problems when interacting with Motibot, particularly when arranging the next session. However, this issue was addressed in the study. Nonetheless, users felt *involved* and *engaged* with Motibot, reporting a worthwhile and absorbing experience and a *positive* perception of use, stating that Motibot was *very easy to use*.

### Limitations and Future Work

This study has 2 main limitations. First, the small sample size, which was chosen following the proof-of-concept phase related to the ORBIT model, as it allows the inclusion of few participants during the first phases of the design, evaluation, and implementation process [40]. However, this choice does not permit data generalization. Second, a more complex analysis approach concerning text mining on the semistructured interviews, such as supervised or unsupervised learning, could not be implemented owing to the relatively small number of participants and the limited length of the interviews (ie, between approximately 200 and 1000 words each). Future studies should integrate, in the development of a VC, medical factors such as the glycemic levels alongside the main psychosocial aspects, as they interplay with the management of DM and thus are variables worth analyzing. Finally, our future goal is to test Motibot with a larger sample size in a randomized controlled trial to investigate the effectiveness of the psychoeducational intervention in a systematic and controlled manner.

### Conclusions

Motibot was developed through a combination of NLU and handcrafted rules with the aim of delivering a psychoeducational intervention for adults with T1DM and T2DM, which allows them to interact by using both free text and structured dialogue interaction. The results of this study showed positive user experience and engagement. In addition, the findings highlighted the usefulness of interacting with a VC to motivate adults with DM to adopt healthy coping strategies. These coping strategies, specifically related to mindfulness practices, allowed a reduction in anxiety, depression, and diabetes-related emotional distress symptoms, while also improving their well-being. This decrease in psychosocial symptoms and increase in well-being was also maintained at follow-up. VCs have the advantage of scalability, which leads to greater user accessibility, and thus, it is available at any time. Moreover, VCs are deployable to adults with DM who show mild and moderate psychosocial symptoms. In particular, VCs can provide them with valuable support, in combination with a dedicated psychotherapist both in a traditional face-to-face setting or in a digital solution referring to the stepped care model [59].

## Appendix

Multimedia Appendix 1 Mean and SD of the User Experience Questionnaire.

## Abbreviations

AADE: American Association of Diabetes Educators
DM: diabetes mellitus
GAD-7: Generalized Anxiety Disorder-7 Items
ML: machine learning
NLU: natural language understanding
ORBIT: Obesity-Related Behavioral Intervention Trials
PAID-5: Problem Areas in Diabetes Scale–Short Form-5 Items
PHQ-9: Patient Health Questionnaire-9 Items
PSS-10: Perceived Stress Scale-10 Items
T1DM: type 1 diabetes mellitus
T2DM: type 2 diabetes mellitus
TTMC: transtheoretical model of change
UE: user engagement
UEQ: User Experience Questionnaire
UES: User Engagement Scale
UX: user experience
VC: virtual coach
WHO-5: The World Health Organization-5 Well-Being Index
