# Supplementary material for: A Virtual Coach (Motibot) for Supporting Healthy Coping Strategies Among Adults With Diabetes: Proof-of-Concept Study
Source: JMIR Hum Factors. 2022 Jan 21;9(1):e32211. doi: 10.2196/32211 (PMC8817220; doi:10.2196/32211)
Supplement: Multimedia Appendix 1 [file humanfactors_v9i1e32211_app1.docx]

Mean and Standard Deviation of the User Experience Questionnaire.

Table S1. Means and Standard Deviations concerning the positive items of UEQ (N = 13).

| Mean (SD) | | | | | | | |
| --- | --- | --- | --- | --- | --- | --- | --- |
|  | Pleasant | Effective | Profound | Cordial | Comprehensible Language | Empathetic | Attentive |
| 4th Session | 4.17 (0.83) | 3.67 (0.65) | 3.58 (0.79) | 4.83 (0.39) | 4.67 (0.49) | 3.58 (1.08) | 3.83 (0.94) |
| 8th Session | 4.33 (0.65) | 3.83 (0.83) | 3.58 (0.79) | 4.75 (0.45) | 4.67 (0.49) | 3.5 (0.80) | 3.58 (0.90) |
| 12th Session | 4.31 (0.75) | 3.85 (1.07) | 3.7 (0.95) | 4.85 (0.38) | 4.77 (0.44) | 3.62 (1.12) | 3.46 (0.87) |
|  | Motivating | Encouraging | Supportive | Trustworthy | Flexible | Interesting |  |
| 4th Session | 4.08 (0.67) | 4.33 (0.65) | 3.75 (0.75) | 4 (0.74) | 3.58 (1.38) | 4.25 (0.75) |  |
| 8th Session | 3.92 (0.67) | 4.08 (0.79) | 4.17 (0.83) | 3.83 (0.72) | 3.75 (1.14) | 4.17 (0.83) |  |
| 12th Session | 4.23 (0.83) | 4.38 (0.87) | 3.77 (1.01) | 4.15 (0.90) | 3.69 (1.03) | 4.53 (0.88) |  |

Table S2. Means and Standard Deviations concerning the negative items of UEQ (N = 13).

| Mean (SD) | | | | | | | | |
| --- | --- | --- | --- | --- | --- | --- | --- | --- |
|  | Not Effective | Annoying | Not Reliable | Unappealing | Unclear | Complicated | Not Efficient | Too Much Information |
| 4th Session | 2.25 (1.42) | 1.08 (0.29) | 1.5 (1.24) | 2.17 (1.34) | 1.92 (1.08) | 1.33 (0.65) | 1.83 (1.19) | 1.25 (0.62) |
| 8th Session | 1.75 (1.06) | 1.5 (0.90) | 1.83 (1.40) | 1.67 (0.78) | 1.58 (1.16) | 1.42 (0.51) | 1.67 (0.89) | 1.75 (1.22) |
| 12th Session | 1.92 (1.32) | 1.15 (0.38) | 1.54 (0.88) | 1.54 (0.88) | 1.31 (0.48) | 1.15 (0.38) | 1.77 (1.09) | 1.38 (0.65) |
|  | Dissuading | Not Engaging | Not Stimulating | Unpredictable | Not Reflective | Conventional | Rigid |  |
| 4th Session | 2.5 (1.31) | 2.33 (1.50) | 2.25 (1.22) | 2.08 (1.16) | 1.83 (0.83) | 2.92 (0.90) | 2.67 (1.07) |  |
| 8th Session | 2.25 (0.97) | 1.67 (0.89) | 2.17 (1.19) | 2.08 (0.90) | 1.92 (1.08) | 2.42 (1.24) | 2.42 (1.44) |  |
| 12th Session | 2.31 (1.18) | 1.69 (1.18) | 1.62 (0.87) | 2.15 (0.90) | 1.77 (1.17) | 2.69 (1.25) | 2 (1.00) |  |
